# The Quality of Logistics Management Information System and the Availability of Tracer Drugs at Health Posts in Rural Ethiopia: A Mixed-Method Study

**DOI:** 10.4314/ejhs.v33i1.4S

**Published:** 2023-04

**Authors:** Bisrat Fantaye Denberu, Merga Belina, Mekdes Demissie, Netsanet Abera, Girmay Medhin, Alula M Teklu, Melody Kelemu, Yibeltal Kiflie Alemayehu

**Affiliations:** 1 Federal Ministry of Health, Ethiopia; 2 Addis Ababa University, Department of Statistics; 3 Centre for Innovative Drug Development and Therapeutic Studies for Africa (CDT-Africa), College of Health Science, Addis Ababa University; 4 College of Health and Medical Sciences, Haramaya University, Ethiopia; 5 Hawassa University, School of Public Health; 6 MERQ Consultancy PLC, Addis Ababa, Ethiopia; 7 Addis Ababa University, Aklilu Lemma Institute of Pathobiology; 8 International Institute for Primary Health Care-Ethiopia (IIfPHC-E); 9 Department of Health Policy and Management, Jimma University, Jimma, Ethiopia; 10 Department of Global Community Health and Behavioral Sciences, School of Public Health and Tropical Medicine, Tulane University, New Orleans, USA

**Keywords:** pharmaceuticals, logistics management information system, primary health care, health posts, availability, tracer drugs, Ethiopia

## Abstract

**Background:**

Proper implementation of the logistics management information system (LMIS) would facilitate access to essential pharmaceutical products. It also prevents wastage at health posts. The aim of this study was to assess the implementation of the LMIS and the availability of tracer drugs at health posts in rural Ethiopia.

**Methods:**

We employed a cross-sectional descriptive design with a mixed-method approach. The data used for this paper was collected from March to May 2019 as part of the National HEP assessment. The study involved 343 health posts randomly selected from nine regions of Ethiopia. Women's Development Army members and household heads participated in the qualitative study (i.e. in FGD and KII). The quantitative data were exported from Open Data Kit (ODK) to Stata 15.1 for statistical analysis, and the qualitative data were entered into NVivo 12 and analyzed using thematic content analysis.

**Results:**

Of the health posts, 59.4% had a space for storing drugs; less than half (41.9%; 95% confidence interval (CI) [36%, 48%]) had a functioning refrigerator. The mean percentage of the availability of selected tracer drugs at health posts was 59.6%, with a 95% CI (58.9%, 60.3%). Bin cards were available at 43% (95% CI [40%, 46%]) of health posts, and among these, only 27.5% of the health posts adequately used the bin cards.

**Conclusion:**

The absence and poor use of LMIS tools was observed at health posts. Proper implementation of the LMIS has the potential to improve the availability of essential drugs that, in turn, improve health post level delivery of health services.

## Introduction

The Ethiopian Health Extension Program (HEP) was designed to improve the health of families, with their full participation, using local technologies and the community's skills and wisdom (1). Primary health care (PHC) facilities in Ethiopia obtain pharmaceuticals mainly from the Ethiopian Pharmaceutical Supply Agency through the Integrated Pharmaceuticals Logistics System via consumption-based requests. This approach is believed to ensure an adequate supply of medicines required for the treatment of diseases affecting most of the country's population (2,3).

The primary purpose of a logistics management information system (LMIS) is to collect, organize, and report information to other levels in the system to facilitate decision-making that governs the logistics system (4). The provision of promotive, preventive, curative, and rehabilitative health services depends on the regular availability of relevant medicines of proven safety, efficacy, and quality at an affordable price, and on their proper use (5). There are 17,162 functioning health posts (HPs) in Ethiopia, and to date, about 40,000 health extension workers (HEWs) have been deployed in these HPs. To get meaningful return from this investment at primary health care by improving health of people, the LMIS should be effective and efficient. Ineffective management of the LMIS could lead to stock out of essential drugs and supplies, potential wastage and loss of health pharmaceutical commodities (6,1).

It is unclear whether HPs situated in rural kebeles with limited all wealth transpiration facilities have the necessary resources and tools to implement the LMIS and whether they are making the essential medicines available to their catchment populations. Few studies conducted on the HEP have described LMIS implementation and the availability of drugs at HPs (8,1,9). The limitations of these studies are that one is done in urban HEP setting, and none of them touches the LMIS implementation at HP level. The purpose of this study was to assess whether HPs are properly implementing the LMIS and to investigate the availability of essential tracer drugs for the provision of selected curative and preventive services at rural HPs.

## Materials and Methods

**Study Area**: While the data collection was taking place, Ethiopia was administratively divided into nine regional states (Tigray; Afar; Amhara; Oromia; Somali; Benishangul-Gumuz; Gambela; Harari; and Southern Nations, Nationalities, and People's Region [SNNPR]) and two city administrations. HP is a PHC delivery unit structured to serve 3,000–5,000 people and designed to be staffed by an average of two HEWs (10,11).

**Study design and study period**: This study is part of the national survey that evaluated the Ethiopian HEP, for which data was collected from March 2019 to May 2019 (12). We used a cross-sectional descriptive design with a quantitative and qualitative exploratory approach, nested within this national survey, to assess LMIS practices and the availability of tracer drugs at HPs in Ethiopia.

**Source population**: All HPs in the country constitute the source population for the quantitative assessment. Women's Development Army (WDA) members and heads of household (men and women) residing in the study HP catchment kebeles, HEWs, HC staffs constituted the source population for the qualitative assessment.

**Study participants**: The study HPs for the quantitative part of the assessment were HPs in six randomly selected kebeles within each study Woreda. Health centers (HCs) designated to provide supportive supervision for the study HPs was also investigated. Sixty-two woredas were initially selected using stratified random sampling, with the strata created by combining the nine regional states. Participants in the qualitative study were purposively selected from the study kebeles. These include: HEWs working in the HPs selected for the study, HC staff, WDA leaders (women) and female or male heads of household.

**Inclusion criteria**: All HPs located in the study kebeles and that had been serving the community for at least one year were eligible for assessment. HCs designated to provide supportive supervision for the study HPs were also eligible for inclusion. For the qualitative interviews, which were conducted at community level, HEWs working in the HPs selected for the study, HC staff assigned to supervise the study HPs, WDA leaders who lived in the study kebeles and had held their positions for at least six months, and men and women who were heads of household in the study kebeles and at least 15 years of age were eligible for inclusion.

**Sample size determination and sampling strategy**: The number of HPs required for the HP assessment was calculated taking the best available estimates for selected indicators from the 2016 Service Availability and Readiness Assessment (11). The sample size was calculated with a 95% confidence interval and a 5% margin of error (d = 5%) for HP-level variables, the availability of basic equipment among HPs (57%), and the percentage of HPs with a nonzero stock of oral rehydration solution (ORS; 40%), providing FP services (95%), and with at least one staff member trained to diagnose and treat malaria (47%). Staff trained in diagnosing and treating malaria yielded the maximum sample size of 384 HPs.

To attain the 384 HPs, 64 woredas were selected from nine regional states using a multistage random sampling technique, and from each of these woredas, six kebeles were again randomly selected. We hoped each kebele would have one HP. Next, an HP located in the selected kebele was considered as the study HP. For the qualitative study, a purposive sampling technique was employed to identify individuals for key informant interviews (KIIs) and focus group discussions (FGDs). HCs that supervise the study HPs were also included in the assessment.

**Data collection tools and procedures**: A structured questionnaire adapted from the Logistics Indicator Assessment Tool developed by DELIVER (13) was used to collect quantitative data from the HPs and supervising HCs. The assessment was done by interviewing the heads of the HPs, making observations in the HPs, and reviewing HP records (files, logistics forms, registers, and reports).

Semi-structured qualitative survey tools were translated into six local languages (Amharic, Afan Oromo, Tigrigna, Somali, Nuer, and Afar) and then translated back into English to ensure accuracy. Trained data collectors collected the data through FGDs and KIIs. Each FGD had six to eight participants, and KIIs were conducted at HC, heads of the household. KII topic guides were used to guide the interviews. The audio from each KII and FGD were recorded to ensure its accurate and complete transcription.

**Quality assurance**: Both qualitative and quantitative data collectors were trained for 10 days. The training sessions covered general guidelines on data collection methods, sampling and data collection procedures, and the contents of each data collection tool. A pretest was done and the necessary corrections made.

### Description of Outcome Variables

**Availability of tracer drugs**: In this study, we used the following ranges (13): ≤ 50% – very low, 51%–65% – low, 66%–80% – fairly high, and 80% – high. We assessed the availability of tracer drugs at HPs based on the above definition of availability.

**Timely reporting of the Health Post Monthly Report and Resupply (HPMRR)**: The date, on which the HPs are expected to submit the monthly reporting form, the HPMRR, is the 1^st^ to 5^th^ day of the current month, were observed. Also, the time of the resupply by the HC was checked to evaluate whether it was within the resupply time. If the resupply of medicines provided by the fifth day of the current month, it is regarded as a timely resupply as reported by the HP.

HCs were also asked whether they resupplied the HP on time. The self-reported response of the HC was considered a timely resupply reported by the HC.

**Data analysis**: All qualitative data were transcribed verbatim and translated into English for coding. The transcripts were compared to the audio recordings to ensure accuracy. Meaningful codes that emerged were grouped into basic themes, allowing main themes to emerge from the data. Qualitative data were entered into NVivo 12 and analyzed using thematic content analysis. Electronically collected quantitative data were exported from ODK to Stata 15.1 for statistical analysis.

**Ethical considerations**: Ethical clearance was obtained from the Institutional Review Board of the Ethiopian Public Health Institute. Study participants were informed about the purpose of the research, the harms and benefits of participation in the study, and their right not to participate in the study and to withdraw at any point in the data collection process, even if they initially consented to participate. Consent was solicited from all participants before the commencement of the interviews. Every effort was made to maintain participants' confidentiality during data collection, analysis, and writing. The privacy and confidentiality of interviewees were protected with codes and pseudonyms when storing data and writing this paper. In reporting the findings, quotations are identified only by source (e.g., KII, FGD), location, and participant group.

## Results

Data were collected from 343 of 384 target HPs. The reasons for non-responses were: Two woredas that would have resulted in 12 additional HPs were not surveyed due to the prevailing security situation during data collection. Moreover, eight kebeles did not have HP. In addition to these, 21 HPs were not functioning during the survey period (*due to damage* or *having been demolished* and there were no HEWs in the HP *HEW*).

### LMIS Practices

**Storage conditions of drugs at HPs**: Only 59.4% of HPs had sufficient space for the storage of drugs and medical supplies. Overall, 79% of the storage areas were protected from sunlight, whereas 63% were clean and ventilated. Less than half of the HPs (41.9%; 95% CI [36%, 48%])) had a functioning refrigerator for storing medicines (vaccines and test kits), and of these, only 77% had a functional thermometer (i.e. an external thermometer used to check storage temperature). Regional disparity was observed in the availability of the necessary equipment for storing medicines. The Amhara region had the lowest number of HPs (22.9%) with functioning refrigerators, and a higher proportion was observed in the Gambela region (85.9%; Table 2).

**Table 2:** Percentage of HPs in rural areas that have storage areas for drugs, and the storage conditions of drugs

Region	Storage area available (%)	Clean (%)	Ventilated (%)	Protected from sunlight (%)	Space sufficient for existing product (%)	Functioning refrigerator (%)	Thermometer (%)
Tigray (n = 32)	61.5	91.4	80.3	89.0	80.0	63.2	80.2
Afar (n = 18)	89.7	94.9	91.8	86.7	71.8	52.0	77.4
Amhara (n = 60)	88.5	63.5	61.0	91.4	42.7	22.9	80.9
Oromia (n = 74)	46.0	50.0	57.6	59.4	15.6	35.6	74.2
Somali (n = 43)	26.1	60.2	75.0	44.0	53.3	54.1	49.9
Benishangul-Gumuz (n = 24)	69.4	72.7	90.1	95.1	80.3	45.0	75.6
SNNP (n = 59)	50.8	47.0	59.4	75.2	51.2	38.4	85.7
Gambela (n = 17)	71.6	75.9	14.1	67.2	53.1	85.9	69.8
Harari (n = 16)	86.5	89.0	83.5	94.5	46.8	73.8	100.0
National (N = 343)	**59.4**	**63.3**	**63.2**	**78.7**	**47.0**	**41.9**	**75.0**

During the FGD, it was noted that the community members observes poor storage of drugs, and they think that this is one of the pushing factors for the community from go to the HP for medical services. Some community members do not go to HP because of lack of sanitation and poor drug handling as noted in this transcript: *The medicines are placed on the floor and they are not placed in an appropriate place…. The community says “the HEWs were sitting in the dust, the drugs in the HPs are also affected by the dusts… (Oromia; FGD with Female Community Members)*.

During the assessment, 143 HPs had two or more expired drugs, and 62.9% adhered to the FEFO arrangement. There are regional differences within this practice: SNNPR had the lowest proportion of FEFO-implementing HPs (10.6%), whereas Harari (84.4%) and Amhara (81%) had relatively high proportions.

### Presence and use of recording and reporting formats for LMIS in HPs

**Availability and use of bin cards**: The overall availability of bin cards at HPs was low (43%; 95% CI [40%, 46%])), with some degree of regional variability. The availability was lowest in the Somali region (1.45%) and highest in the Tigray region (94.53%). Only 27.5% of HPs used bin cards adequately, with the highest proportion of adequate use in the Tigray region and the lowest in the Somali region (Table 3).

**Table 3:** Mean percentages of availability and use of bin cards in HPs in rural Ethiopia

Region	Bin card availability % (%)	Opening balance (%)			Quantity received (%)			Loss & adjustment (%)			Closing balance (%)		

		*Poor*	*Inadequate*	*Adequate*	*Poor*	*Inadequate*	*Adequate*	*Poor*	*Inadequate*	*Adequate*	*Poor*	*Inadequate*	*Adequate*
Tigray (n = 32)	94.53	8.5	6.9	84.6	8.5	13.4	78.1	11.8	10.6	77.6	15.4	16.4	68.1
Afar (n = 18)	24.14	65.8	0.0	34.2	65.8	6.5	27.7	65.8	6.5	27.7	83.1	0.0	16.9
Amhara (n = 60)	65.75	47.2	5.4	47.5	53.5	4.3	42.2	49.4	4.5	46.1	53.8	3.8	42.5
Oromia (n = 74)	32.76	57.9	16.9	25.3	59.4	8.7	31.9	69.0	12.5	18.5	73.6	9.8	16.7
Somali (n = 43)	1.45	95.7	2.8	1.5	98.5	0.0	1.5	98.5	0.0	1.5	98.4	0.0	1.6
Benishangul-Gumuz (n = 24)	30.59	57.5	25.7	16.8	57.5	10.5	32.1	67.9	0.0	32.1	84.8	4.8	10.5
SNNP (n = 59)	38.33	72.4	18.8	8.9	75.3	18.5	6.3	77.0	13.8	9.2	80.1	15.3	4.7
Gambela (n = 17)	25.97	83.4	11.9	4.7	90.6	4.7	4.7	95.3	0.0	4.7	95.3	0.0	4.7
Harari (n = 16)	86.51	36.5	0.0	63.5	27.8	22.2	50.0	27.8	31.0	41.3	68.3	13.5	18.3
**National (N = 343)**	**43.0**	**59.0**	**11.3**	**29.7**	**61.8**	**8.8**	**29.4**	**64.3**	**8.0**	**27.7**	**68.8**	**7.9**	**23.3**

**Availability and use of HPMRR**: A high proportion of HPs (85.7%) had HPMRR reporting forms, although their use was lower. The proper use of the HPMRR for the resupply of drugs was highest in the Tigray region and lowest in the Somali region (Table 4).

**Table 4:** Availability and use of HPMRR at HPs in rural Ethiopia

Region	HPMRR availability (%)	Opening balance (%)			Quantity received (%)			Closing balance (%)		

		Poor	Inadequate	Adequate	Poor	Inadequate	Adequate	Poor	Inadequate	Adequate
Tigray (n = 32)	100.0	4.6	3.3	*92.2*	4.6	3.3	*92.2*	4.6	0.0	95.4
Afar (n = 18)	100.0	59.3	0.0	40.7	59.3	0.0	40.7	76.6	0.0	23.5
Amhara (n = 60)	94.3	49.8	2.8	47.5	45.1	4.7	50.3	50.9	3.8	45.3
Oromia (n = 74)	74.1	59.7	11.5	28.9	69.4	6.5	24.1	69.3	7.1	23.6
Somali (n = 43)	100.0	94.6	3.9	1.5	94.6	3.9	1.5	98.5	0.0	1.5
Benishangul-Gumuz (n = 24)	75.4	43.3	27.6	29.1	45.4	20.6	34.0	72.3	8.9	18.8
SNNP (n = 59)	66.2	49.6	12.5	37.9	58.3	13.6	28.1	71.0	10.0	19.0
Gambela (n = 17)	81.9	76.5	13.1	10.4	84.4	5.2	10.4	89.0	0.0	11.0
Harari (n = 16)	100.0	50.6	18.7	30.8	31.9	24.2	44.0	78.5	0.0	21.5
National (N = 343)	**85.7**	**54.20**	**8.63**	**37.17**	**57.2**	**7.4**	**35.4**	**63.6**	**4.7**	**31.8**

Note: HPMRR: Health Post Monthly Reporting and Resupply form

**Availability of tracer drugs**: Fifteen tracer drugs were identified from the full list of products managed by HEWs. Among the identified drugs, the availability was lowest for paracetamol syrup or suppositories (18.8%) and highest for ORS (85%) and disposable syringes (85.5%; Table 5).

**Table 5:** Percentage of availability of each tracer drug at HPs in rural Ethiopia

Region	ORS	Pentavalent	Implanon NXT	Depo	Zinc tab	Vitamin A	TTC eye oint.	Amox Dt/Suspension	PCM syrup/sup	Albendazole	PCM tab	Fefol tab	OCP	Artemisinin	Disposable syringe
Tigray	100	41.7	100.0	96.4	96.7	96.4	76.1	56.8	36.4	96.4	83.0	97.7	65.8	66.1	96.4
Afar	72.9	36.8	2.8	92.6	63.0	94.5	63.7	45.8	30.8	63.2	35.6	43.0	53.6	78.9	100
Amhara	98.9	55.7	70.4	96.1	96.0	94.9	32.3	39.2	12.7	90.4	29.7	80.0	68.6	67.4	94.5
Oromia	86.0	41.2	70.2	94.0	85.9	84.3	28.6	32.4	10.9	71.2	36.9	56.6	60.6	28.1	92.1
Somali	60.6	47.7	10.4	17.7	48.6	53.6	52.7	72.8	59.1	55.2	54.5	65.3	25.8	11.0	50.1
Benishangul-Gumuz	90.9	49.1	72.4	78.8	92.0	27.6	22.4	49.3	19.4	39.5	36.1	60.3	44.8	96.6	85.6
SNNP	74.9	37.4	64.5	83.2	80.2	64.8	21.1	25.7	4.7	60.3	34.0	71.1	50.7	29.9	84.4
Gambela	85.9	71.6	4.7	69.3	64.6	42.5	57.5	49.5	14.1	59.9	52.8	36.2	28.3	37.8	69.3
Harari	90.5	73.8	100.0	100.0	81.0	91.3	9.5	58.7	40.5	90.5	73.0	72.2	67.5	45.2	90.5
National	85.0	46.7	60.9	82.2	82.4	75.3	36.0	41.4	18.8	71.2	41.9	68.0	54.4	43.9	85.5

Note: ORS: Oral Rehydration Salt, TTC: Tetracycline, Amox Dt: Amoxicillin Dispersible tablet, PCM: Paracetamol, Fefol: Ferrous with Folic Acid, OCP: Oral Contraceptive Pills

The community also reported that a shortage of FP drugs led to unwanted pregnancies. For example, one FGD participant said: *There is a shortage of Depo; thus, there was unwanted pregnancy…. Due to the shortage of contraceptives women might have unwanted pregnancy. (Amhara; FGD with Male Community Leaders)*.

The finding was further supported by a WDA member and health extension workers, who explained the shortage of supplies in general, noting that it was worst when it came to drugs and pharmaceuticals: *HEWs have a shortage of drugs and vaccines. Until vaccines arrive, the community suffers a lot. Such a shortage of supplies has made the HEP service weak. (Amhara; FGD with WDA Members)*

Another participant said:

*The office of the health extension professional is almost empty. There are a few birth control pills available, but there are no medicines… The only things we get from the HEWs are alcohol for the wounds and that's it. (Amhara; FGD with WDA Members)*.

Health extension workers from Oromia region also said: *We are sending pneumonia, cough, and measles case to HC; we are trained about these diseases but we couldn't provide service due to lack of drug…* (KII with HEW, Oromia region).

**Figure 2:**
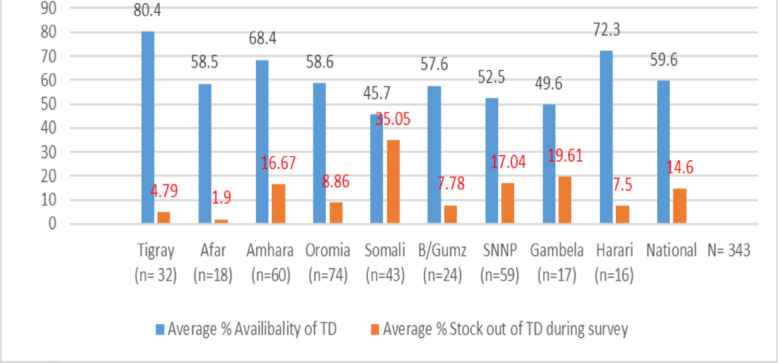
Regional distribution of mean percentage availability and percentage mean stock of tracer drugs in rural Ethiopia

The mean percentage of the availability of selected tracer drugs at HPs was 59.6% (95% CI [58.9%, 60.3%]), and it was lowest in the Somali region (45.7%) and highest in the Tigray region (80.4%). The overall mean stock-out percentage during the preceding six months was 14.6%, with a significant regional difference (Figure 2). The highest mean stock-out was in the Somali region (35.05%), and the lowest was in the Afar region (1.9%).

**Availability of tracer drugs and timely reporting of HPMRR**: In this study, 56.2% of the HPs submitted the HPMRR to the HCs on time for resupply, and only 53.3% received the resupply on time. Based on the responses obtained from catchment HCs, 85.47% of HCs made a timely resupply to the HPs. The responses from HCs contradict the reports from HPs (Table 6).

**Table 6:** Regional distribution of mean percentage availability and timely resupply of medicine by HCs to HPs in rural Ethiopia

Region	Mean availability of TD (%)*	Timely submission of HPMRR to HC (%)	Timely resupply to HP by HC, record verified at HP (%)**	Timely resupply to HPs, data collected at HC (%)**
Tigray (n = 32)	80.4	94.7	82.4	85.2
Afar (n = 18)	58.5	42.8	40.7	85.7
Amhara (n = 60)	68.4	90.5	86.9	94.9
Oromia (n = 74)	58.6	37.9	41.4	93.5
Somali (n = 43)	45.7	23.2	24.5	60.0
B-Gumuz (n = 24)	57.6	71.2	70.1	83.3
SNNP (n = 59)	52.5	38.8	29.8	68.6
Gambela (n = 17)	49.6	66.2	57.5	100.0
Harari (n = 16)	72.3	63.5	58.7	100.0
National (N = 343)	**59.6** *	**56.2**	53.3*	**85.5**

Note. TD: tracer drug; HPMRR: Health Post Monthly Reporting and Resupply form; HC: health center; HP: health post; B-Gumuz: Benishangul-Gumuz; SNNP: Southern Nations, Nationalities, and Peoples' Region

* Mean percentage availability of TDs is correlated with the timely resupply of the HP by the HC as per the HP report. The correlation proportion was found to be strong at 74.64% (P = 0.021).

** Timely resupply of medicines by HC reported by HP (53.3%) and timely resupply reported by HC (85.5%) were found to differ and are statistically significant (P = 0.009).

An FGD participant complained about HPs, saying HPs were established but were not fully supplied with the necessary materials: *There is a shortage of medicine in health posts. When we finish the first dose and go back for an additional dose, there is no medicine in the health post. Why don't the governments make the health post give a proper service to the people? (SNNP; FGD with WDA Members)*

Head of a health center from SNNP also mentioned the problems as follows: *There are no adequate medical supplies in health posts and health center. HEWs are not fully addressing health problem of the community. In the last six month there were shortages of vaccines so children are not getting immunization. The community is complaining this problem to the kebele. The supply is not consistent. For example, most women want to have the short acting family planning, but it is not available both at health post and health center. SNNP, KII with Head of HC)*.

## Discussion

Most HPs had storage areas for health products, but only 41.9% had functioning refrigerators to store vaccines and test kits. The use of the necessary LMIS features such as bin cards and HPMRR was also poor. Only a quarter of the HPs were adequately using the features for requesting the resupply of pharmaceuticals. The mean availability of tracer drugs was also low.

Some HPs did not have a space for storing drugs and medical supplies. In line with the current findings, the major technical challenges expressed by HEWs regarding the implementation of the HEP were the absence of storage and carriage for vaccines (1). Less than half of the HPs visited during the survey had a functioning refrigerator for storing vaccines and test kits. Vaccine potency can diminish when they are exposed to inappropriate temperature, and cannot be regained; thus, the vaccines must be protected from temperature extremes (14). Most HPs in the country face challenges in storing vaccines under conditions that meet requirements.

FEFO implementation practices were found to be fairly good, as 62.9% of the HPs adhered to the FEFO method, which is a similar finding to a study conducted in Kenya (15). Higher FEFO adherence in health facilities (HFs) was previously reported in South West Ethiopia(16) and Addis Ababa (3). This higher percentage could be due to the number and tier levels of facilities included in those studies. Failure to observe the expiry dates of medicine could lead to the loss of a significant amount of resources (17). One of the contributing factors for medicine wastage in public health facilities is not adhering to the FEFO principles of store management (18).

The availability of standard logistics recording and reporting tools or formats and the proper use of these tools play a substantial role in the implementation of effective and efficient LMISs (19). The overall availability of bin cards in HPs was low, with significant regional variability. As part of the PHC unit, HPs manage pharmaceuticals that are used at the community level, and one of HPs' major responsibilities is to maintain bin cards for all pharmaceuticals (4). However, our findings indicate low use compared with a study conducted in Addis Ababa (3). The observed difference can partly be explained by the settings of the HFs and partly by the tier levels of HFs, as the HCs in Addis Ababa are higher tier and better situated than the rural HPs included in the current study.

The use of both important LMIS tools (bin cards and HMPRR forms) in the HPs was low. All HPs are expected to fill out and complete the HPMRR form monthly (4). However, our findings show that less than half of the HPs adequately used these tools. Updating bin cards and adequately using HPMRR forms the basis for the resupply of drugs from the higher tier institution, which is the HC in rural Ethiopia. Adequately filling out the bin cards and submitting the HPMRR form in a timely manner will positively influence the availability of drugs at a HP. Hence, the absence and improper use of the LMIS tools will affect the efficient use of the pharmaceutical logistics system. The absence of an important LMIS tool and poor use of the available tools could lead to the inefficient implementation of the logistics system, which could directly contribute to the wastage of pharmaceutical resources

A sustainable supply of essential medicines is required to avoid medicine shortages that can cause avoidable suffering and death (20). In this survey, a significant proportion of HPs did not have important tracer drugs and would be categorized as “low availability” according to WHO recommendations (20). The overall mean availability of tracer drugs in the current study is much lower than the mean availability reported in 2012 in the Amhara region (21) and somewhat better than the mean availability reported in the Tigray region in 2016 (22). Timely resupply to the HP by the HC, data that were obtained by reviewing HP documents, are strongly and significantly correlated with the mean availability of tracer drugs. The supplying HC failing to calculate the issue quantities for each health product reported by the HPs and to resupply the products on time defeats the purpose of the HPMRR and will lead to a shortage of tracer drugs.

This study showed that most HPs lack a functioning refrigerator to keep vital vaccines and test kits at the HPs, which implies the use of medicines stored at below-standard conditions. It also indicated shortage of essential tracer drugs at HPs affects health care service provision in rural communities, where most of the population lives.

As studies on pharmaceutical logistics in HPs are often overlooked, this study provides national evidence on LMIS practices and the availability of tracer drugs at HPs. However, because this study was nested within a national survey, it could not assess the specific management support provided to HPs for the implementation of LMIS tools.

## Figures and Tables

**Table 1:** Description of variables and definition of LMIS tool use

Variable	Response categories	Definition
Bin card	Used poorly	No entries
	Used inadequately	Four entries fully completed on one or two cards
	Used adequately	Four entries fully completed on all cards
HPMRR	Used poorly	No entries
	Used inadequately	One, two, or three entries
	Used adequately	Four or five entries
Entries in HPMRR assessed during survey	Opening balance Quantity received Closing balance	Loss and adjustment column omitted from assessment, as it was empty at all pretested HPs
FEFO method used	Yes	HP had FEFO arrangement for three randomly picked bin cards for two or more expiry dates
	No	HP did not to arrange drugs using FEFO method for any of the three randomly picked bin cards

*Note:* A bin card is a form in the LMIS used to track stock in storage. It should be kept with the pharmaceutical item. Four entries were checked on three cards selected at random

HPMRR: Health Post Monthly Reporting and Resupply form; three entries on five months' HPMRR forms were observed during the assessment.

FEFO: first to expire; first out; the FEFO method of the arrangement of drugs was checked by picking three bin cards of drugs that have two or more expiry dates and cross-checking their arrangement on the shelves.

HP: health post

**Figure 1:**
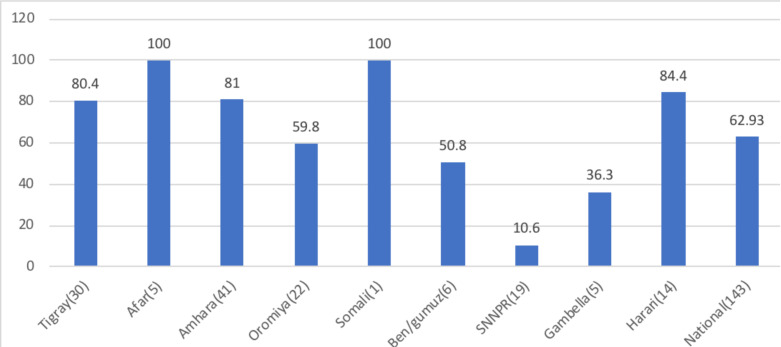
Regional distribution of the percentage of HPs adhering to FEFO storage
